# Task-Dependent Visual Topographic Connectivity in the Human Cerebellum

**DOI:** 10.1523/JNEUROSCI.1370-23.2025

**Published:** 2025-11-25

**Authors:** Wietske Zuiderbaan, Wietske van der Zwaag, Tomas Knapen

**Affiliations:** ^1^Spinoza Centre for Neuroimaging, Amsterdam 1105 BK, Netherlands; ^2^Computational Cognitive Neuroscience and Neuroimaging, Netherlands Institute for Neuroscience, Royal Netherlands Academy of Sciences, Amsterdam 1105 BA, Netherlands; ^3^Department of Radiology and Nuclear Medicine, Amsterdam University Medical Center, University of Amsterdam, Amsterdam 1105 AZ, Netherlands; ^4^Behavioral and Movement Sciences, Vrije Universiteit, Amsterdam 1081 BT, Netherlands

**Keywords:** cerebellum, connectivity, natural scenes, visual system, visual topography

## Abstract

The role of the cerebellum has long been thought to be limited to sensorimotor processes. Recently, its involvement in a broader set of cognitive and associative tasks has challenged this view. Recent studies have expanded the cerebellum's functional repertoire into the visual domain, by identifying three topographically organized clusters exhibiting visual spatial responses using the population receptive field model. In this experiment, researchers used a simple retinotopic mapping stimulus during strict fixation. This represents a situation very different from our everyday vision, which is characterized by continual eye movements, and complex naturalistic visual stimulation. This makes it hard to translate the previous results to natural, active vision. Here, we used topographic connectivity from V1 to investigate the visual topographic organization in the human cerebellum (of either sex) and its dependence on cognitive state (comparing movie watching and resting state experiments). We find that movie watching evokes visual representations with a clear eccentricity gradient in OMV that was not found in a simple retinotopic mapping experiment. We furthermore discovered a novel topographically organized area in the cerebellum, again evoked specifically during movie watching and not in resting state. This latter area is located in the cerebellar Crus II area and falls within regions usually assigned to the cerebellar default mode network. Our results show that we can reveal task-dependent properties of the visual organization when using different cognitive states and how this can provide information about the processing of visual information, also in regions not previously considered to be visually responsive.

## Significance Statement

We used the connective field (CF) model to investigate topographic visual organization in the cerebellum and its dependence on cognitive state. Previous work found three clusters with topographic maps of visual space in the cerebellum. The CF model allowed us to first reproduce and then extend these maps, and study task effects on the visual topographic organization of the human cerebellum. First, we demonstrate previously unseen visual field eccentricity gradient in the oculomotor vermis, present only during naturalistic movie watching. Furthermore, naturalistic movie watching uncovered an additional topographically organized cluster in Crus II. These results show that the topographical organization is flexible across tasks and underline the importance of using naturalistic stimuli to probe high-level visual topographic function.

## Introduction

A growing body of research shows that the cerebellum plays a role in the processing of visual information (Brissenden et al., 2018; van Es et al., 2019; Xue et al., 2021). Both resting state and task-based neuroimaging studies found the cerebellum to be involved in visuospatial cognition/attention and eye movements (Buckner et al., 2011; Voogd et al., 2012; Brissenden et al., 2018; King et al., 2019). Ipsilateral coverage of visual space was found in lobule VIIb/VIIIa of the cerebellum (Brissenden et al., 2018), and nodes in the cerebellum show functional connectivity to retinotopic areas in cerebral cortex, including early visual cortex (Xue et al., 2021).

In cerebral cortex, visual field maps are organized retinotopically (Wandell et al., 2007), i.e., neurons are organized according to the spatial arrangement of the retina. Recently, van Es showed that the cerebellum contains five topographically organized maps with similar properties as visual field maps in cerebral cortex (van Es et al., 2019). The visual nature of these regions was identified using population receptive (pRF) modeling, which estimates for every cerebellar voxel the region of visual space that it responds to. The pRF model is usually estimated using a standard bar mapping stimulus. During the experiment, subjects are required to maintain fixation at the center of the screen. These stimuli lack an important amount of higher-level information as it is typically encountered in real life, and the use of a fixation task limits ecological validity. Therefore, the bar mapping stimulus may not drive high-level visual processing as strongly as naturalistic stimuli would. Moreover, the cerebellum's role in sensorimotor integration may mean that different cerebellar regions are visually responsive, especially during active, naturalistic vision.

Here, we study the visual topographic organization of the cerebellum under more naturalistic conditions. We used data from the human connectome project (HCP) of both movie watching and resting state experiments (Van Essen et al., 2013). This dataset provides a publicly available high-resolution 7T dataset from a large number of participants and therefore gives us the opportunity to study the cerebellum that has a relatively low BOLD signal-to-noise ratio (SNR; Pfaffenrot et al., 2018; Priovoulos et al., 2023).

We analyzed these datasets using connective field (CF) modeling (Haak et al., 2013; Knapen, 2021; Tangtartharakul et al., 2023), which uses topographic functional connectivity to find evidence of visual spatial processing. The advantage of this technique is that it can be used for any type of stimulus and therefore allows us to compare the topographic organization among different cognitive states.

The CF model fitting procedure estimates, for every voxel in the cerebellum, the parameters of its best-fitting CF. This is a Gaussian patch on the surface of V1, with a specific location on the V1 surface and a spatial spread. As the V1 surface orderly represents the visual field, we can translate V1 location to visual field position. This means we can derive the position in visual space that any cerebellar voxel responds to.

Comparing position preferences in the cerebellum between cognitive states, we find differences that are particularly striking in the movie watching experiment. In region OMV, which is known for its role in the making of eye movements (Voogd et al., 2012; King et al., 2019), we find a progression of eccentricity along the cerebellar surface in the MW experiment that was not found previously using the standard bar mapping stimulus. Furthermore, we find a new area in Crus II of the cerebellum to reveal properties of a visual topographic area only during movie watching. This region was not found to be visually responsive before. Interestingly, it is part of the default mode network and shows properties similar to previously found topographic areas in the cerebellum. We show that the CF model allows us to study how the cerebellum encodes spatial information among different experiments. The use of different cognitive states with more naturalistic experiments reveals information about how and which stimulus properties are processed in a certain region.

## Materials and Methods

### Data

For our analysis, we compared visuospatial organization in the cerebellum, as derived from topographic functional connectivity, for different cognitive states. We used the publicly available 7T fMRI dataset from the Human Connectome Project (HCP) that contains Movie Watching (MW), Resting State (RS), and pRF mapping experiments (Van Essen et al., 2013). We used the data of 174 subjects (of either sex), where for every subject there was ∼1 h of data for MW and RS each and ∼30 min of data for the retinotopy experiment. In the MW experiment, subjects viewed a concatenated movie consisting of short fragments of Hollywood movies and independent films and were allowed to make eye movements. In the RS experiment, subjects had their eyes open and performed a fixation task without further visual stimulation. All subjects had normal or corrected-to-normal visual acuity. The data was acquired at a spatial resolution of 1.6 mm^3^, with a time repetition (TR) of 1 s. We used the functional data in combination with the 3 T anatomical MRI images, with subcortical regions in volumetric MNI space and left and right hemispheres sampled to the average-subject surface, aligned using HCP's MCMAll intersubject alignment. For a more detailed description of the data, see Van Essen et al. (2013).

### Analysis

**Figure 1.**
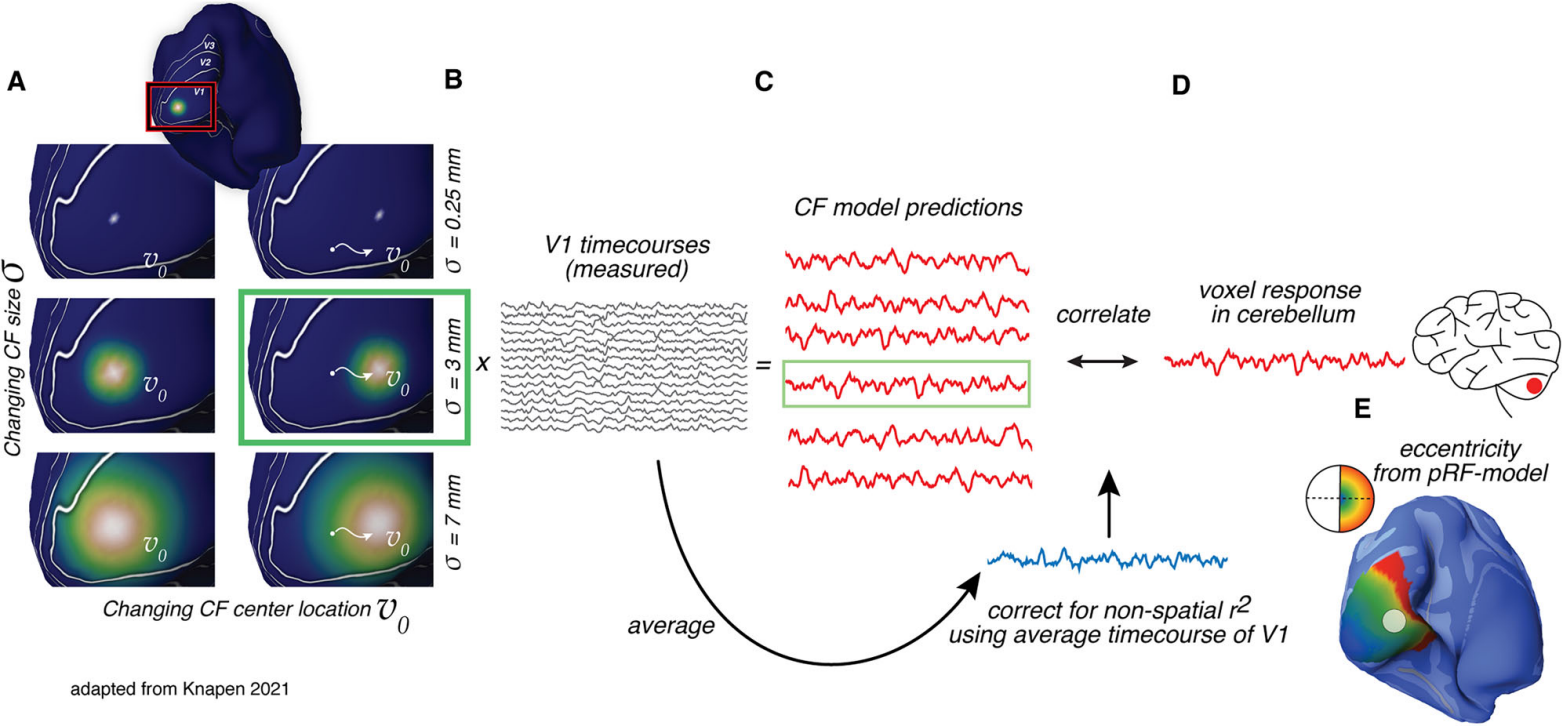
Connective field mapping (CF; Haak et al., 2013; Knapen, 2021; Tangtartharakul et al., 2023). A CF is a Gaussian patch on the V1 surface, with position and size parameters. CF model predictions (***C***) are made for many CFs with different parameters (***A***) from the CF-weighted sum of the V1 timecourses (***B***). The CF model that shows the highest correlation with the measured cerebellar voxel timecourse is selected as the CF of that voxel (green box). Visual field position parameters of the CF are derived from the underlying pRF parameters (***E***).

For our analysis we used the CF model. More details of this technique can be found in Haak et al. (2013) and Knapen (2021), but we will include a brief explanation here:

The CF model combines the pRF model (Dumoulin and Wandell, 2008) with functional connectivity (Fig. 1). First, the pRF model estimates for every voxel in V1 the region of visual space that it responds to, this is the voxel's pRF (Fig. 1*E*). Second, the CF model projects the canonical visual field map of V1 into the cerebellum. It does this by estimating for every voxel in the cerebellum a best-fitting connective field (CF): this is a Gaussian patch on the surface of V1 (Fig. 1*A*). A model timecourse prediction for a CF (Fig. 1*C*) is made from a weighted sum of the CF with the underlying timecourses of the voxels in V1 (Fig. 1*B*). The CF model parameters are optimized as follows: we performed a grid search across peak location and size parameters of the CF, choosing the parameter combination that gives the highest correlation with the predicted and the measured voxel time course in the cerebellum (Fig. 1*C*,*D*). Candidate peak positions on the V1 surface were limited to the set of vertices with an eccentricity of <5° of visual angle and an explained variance of >20%. Where the CF model parameters were estimated per individual subject, the pRF analysis was based on the averaged across-subject timecourses of the HCP retinotopic mapping experiment (Knapen, 2021). The retinotopy of visual area V1 is similar across subjects (Benson et al., 2022), and by averaging the V1 timecourses, the SNR is increased to provide a very robust and high-quality map of V1. The conservative cutoff for eccentricity was used to ensure that we used reliable pRF estimates by using voxels that are visually responsive to a region inside the stimulus range. This improves the interpretability of the inferred spatial preferences of the CF. To ensure that the CF model represents a spatially specific location in V1, the model was corrected using a null model, which is the average timecourse of V1 (Fig. 1*B*,*D*). This null model corrects for responses that are driven by arousal, overall contrast or feature energy, or other global factors that may drive functional connectivity. The performance (topographic improvement) of the CF model is represented in corrected *R*^2^ values; this is the difference (Δ*R*^2^) between the *R*^2^ of the topographic model and the *R*^2^ of the null model. Last, we derived for every CF the parameters for visual field position (*x*,*y*) from the underlying pRFs of the CF, and from these values, we calculated the corresponding eccentricity values at a per voxel basis (Fig. 1*E*). When using average eccentricity values over subjects, we first averaged *x* and *y* parameters over subjects per voxel, and from these average values, we computed the eccentricity values.

### Averaging and thresholding of the data

CFs were fit for individual participants (*N* = 174) using a leave-one-run-out cross-validation procedure. The model was fitted on three runs and tested on the remaining run. The parameters of the CFs were first averaged per subject over the four cross-validated runs. Then we averaged the CFs over subjects. For averaging the parameters of *x*,*y* and eccentricity over subjects, we used a threshold on the performance of *R*^2^ > 0.01 for individual subject data, and after this, a threshold of *R*^2^ > 0.025 for data averaged across subjects. Both thresholds were performed on a per-voxel basis, so no subjects were entirely excluded from the analysis. To obtain this threshold on performance of the CF model, we performed a *t* test (over subjects) on the topographic improvement (Δ*R*^2^) for all the voxels of the cerebellum. The value of 0.01 denotes a minimum *t* value of ∼2 and an average *t* value of ∼3.3. We used both thresholds to exclude unreliable CF estimates.

After averaging the data, we masked out cerebellar voxels that neighbor the cerebral visual cortex, at the superior surface of the cerebellum (lobules V-VI/Crus I). This is necessary, as partial voluming can cause these signals from cerebral visual cortex to erroneously appear in cerebellar voxels (van Es et al., 2019). We used the mask that was used previously (van Es et al., 2019), where they selected voxels based on their location between the cerebrum and cerebellum, together with a stark deviation of pRF parameter values.

### Visualization of parameters on the flatmap

To visualize the parameters of the CFs in the cerebellum, we used the spatially unbiased atlas template (SUIT; Diedrichsen and Zotow, 2015). This toolbox projects the estimated parameter values of the CFs from volumetric MNI space onto a flattened 2D representation of the cerebellum.

### Regions of interest

To compare the performance of the CF model for MW and RS in different regions of the cerebellum, we used as our regions of interest (ROIs) both the topographic areas in the cerebellum as defined by van Es et al. (2019) and the seven functional resting state network ROIs defined by Buckner et al. (2011). These ROIs were available in MNI space, and we resampled these ROIs to the spatial resolution of the functional data, 1.6 mm isotropic.

### Eccentricity gradients

We compared the eccentricity gradients (progression of eccentricity values along the cortex) in the previously defined topographic areas as they were measured using the pRF model (van Es et al., 2019) with the eccentricity gradients found using the CF model. For this, we used the direction of the projection as previously indicated by van Es et al. (2019). The lines of the eccentricity gradient in the CF model (Fig. 3*B*) reflect the mean eccentricity of the average HCP subject binned across each decile of the vertices on the projected surface.

### Plotting of visual field positions

To further investigate the positions of the CFs in the visual field independent of their point of reference, we plotted the positions of the CFs in the visual field using a color that was defined by their positions on the cerebellar surface. This shows how the position in the visual field is dependent on the position along the cerebellar surface. Both horizontal and vertical directions of the ROI are represented by a change in the color axis. The horizontal axis is defined by a change in color from red to green whereas the vertical axis is represented by the intensity of the blue color channel.

### Statistics

We performed *t* tests, where subjects were treated as individual observations, to test for significant differences between performance of RS and MW in the different ROIs in volumetric space. For comparison of the model performance between the experiments, we did not use a threshold on performance.

The stability of the eccentricity gradients was illustrated using a repeated (10.000×) split-half analysis across subjects, where different subsets of the population (half) were made using different permutations of subjects (Fig. 4*C*).

We retested the preferences of lower visual field bias (*y*) and ipsilaterality (hemispheric preference) as they were found in the previously defined topographic areas (van Es et al., 2019) for the CF models that were estimated on RS and MW data using a *t* test.

A difference in eccentricity preference between the pRF model and the CF model estimated on MW and RS data was tested using a *t* test. Here we performed a *t* test over the difference of mean eccentricity values between the CF model (individual eccentricity value per subject) and the mean eccentricity value of the pRF model (average subject) in region OMV.

Statistics for the preference of visual field position in up/down (*y*), left/right (*x*), and ipsilaterality (hemispheric preference) between the left versus right ROIs in the new topographic area were performed using a *t* test.

## Results

### Performance of the model fits

**Figure 2.**
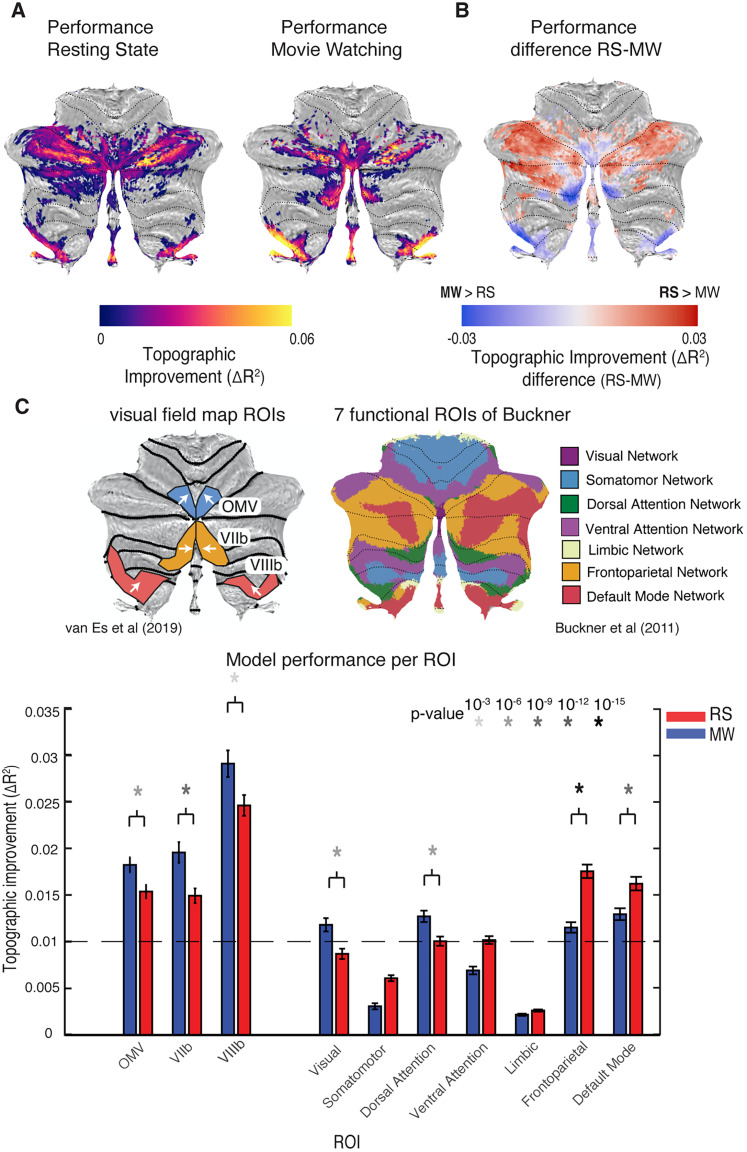
Model performance for RS and MW projected on the SUIT template (***A***). ***B***, Performance difference for RS and MW. Blue regions in ***B*** are the areas where MW outperforms RS; we see that these regions correspond to areas identified in retinotopic mapping (***C***). ***C***, Model performance averaged over ROIs. The error bars reflect the standard error of the mean.

The CF model gives a model prediction of the timeseries per voxel in the cerebellum. We compared the performance of the model fits between experiments: resting state (RS) and movie watching (MW). For this, we used the cross-validated (CV) prediction performance that was corrected for the prediction performance of a nontopographic null model: the average time course of V1. First, we visualized the performance of both CF models for RS and MW projected on the cerebellar surface (Fig. 2*A*). We only plotted the values for which the average topographic improvement (Δ*R*^2^) over the null model was larger than 0.025. This threshold is surpassed in a variety of cerebellar regions, indicating that these regions enjoy topographically specific functional connectivity with early visual cortex.

To compare the performance between the experiments per location on the surface, we subtracted the null-corrected performance of MW from that of RS (Fig. 2*B*). The blue color indicates that MW explains more variance than RS, whereas the red color indicates that RS performs better. We see that in the previously found topographically organized areas (Fig. 2*C*), the fits for the MW experiment explain more of the signal variance than the RS experiment fits, a pattern of results corroborating the visually driven nature of topographic connectivity in these regions. We quantified these observations for the topographic areas defined by van Es et al. (2019), and for the seven functional ROIs of Buckner et al. (2011).

For this, we plotted the average topographic improvement (Δ*R*^2^) per ROI and performed a *t* test over the performance of MW and RS per ROI. Statistics for the topographic ROIs show a significant improvement for MW in all the topographically organized areas [*t*_(173)_ = 4.59, *p* << 0.001 (OMV), *t*_(173)_ = 6.16, *p* << 0.001 (VIIb), *t*_(173)_ = 3.70, *p* < 0.001 (VIIIb)]. Statistics for the seven RSN-based ROIs of Buckner et al. show a significant difference in performance between WM and RS for four ROIs. We only tested the ROIs where at least one model was able to explain the data significantly (Δ*R*^2^) average above 0.01, indicated with the striped line in the figure [*t*_(173)_ > 5.40, *p* << 0.001 (VIS, DORS, FP, and DM)].

### Eccentricity progression, ipsilaterality, and lower visual field bias in the topographic areas

**Figure 3.**
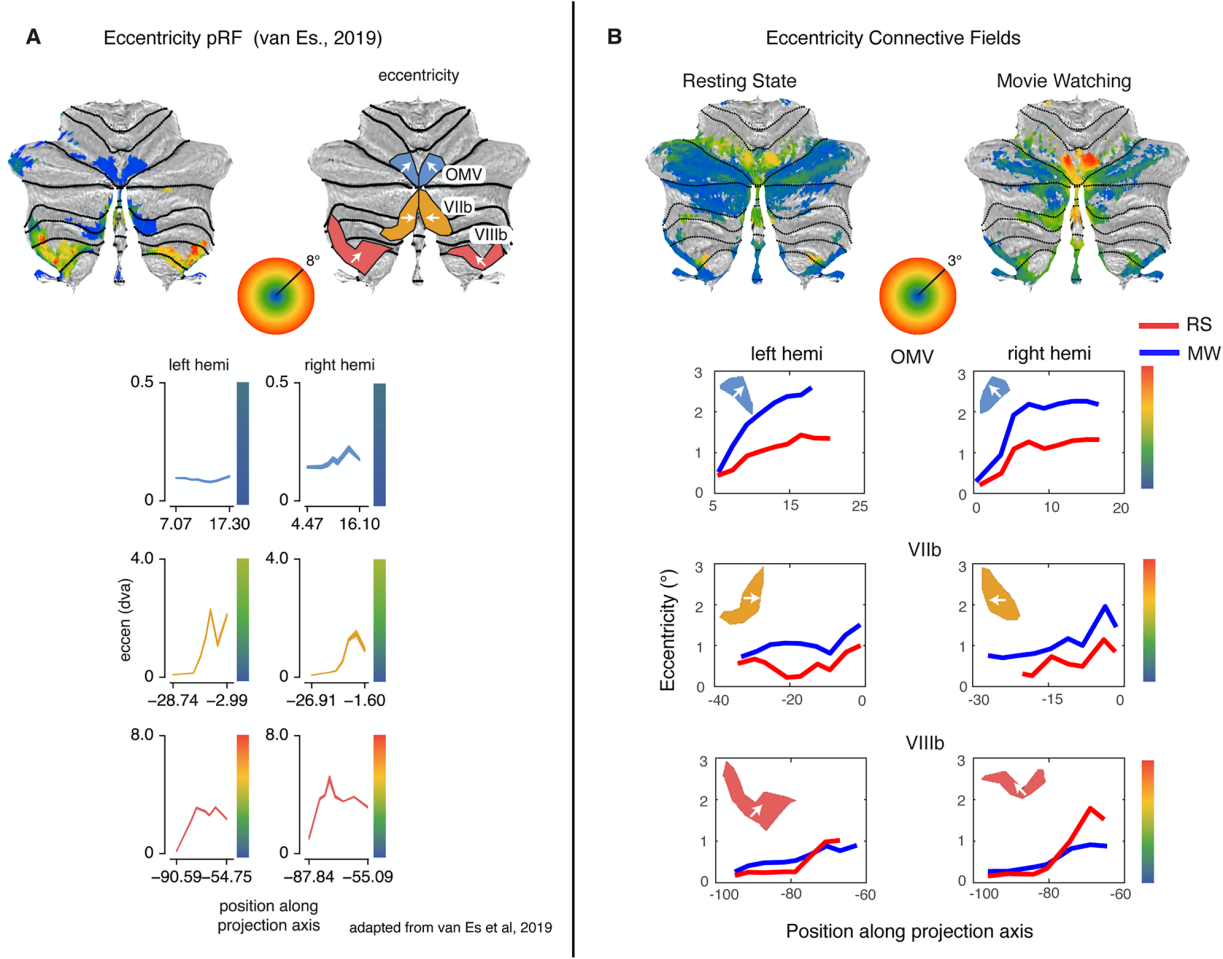
***A***, Eccentricity progression as found with the pRF model (van Es et al., 2019). Line plots average eccentricity measures in the direction of the arrow and reflect the mean eccentricity of the average HCP subject binned across each decile of the vertices on the projected surface. ***B***, Connective field map eccentricity for both MW and RS. OMV (top row) sees, especially for MW, an increase in the progression of eccentricity in ***B***, but not in ***A*** (blue line). Clusters in lobules VIIb (middle row) and VIIIb (bottom row) show comparable eccentricity progressions in ***A*** and ***B***.

In the study of van Es et al. (2019), a gradient of eccentricity preference was quantified on the cerebellar surface for every topographic region. The direction of this eccentricity gradient is indicated with the white arrows on the surface (Fig. 3*A*). In Figure 3*B*, we show the line plots for the eccentricity gradient in the CF model along the same direction, indicated on the surface for both MW (blue line) and RS (red line) experiments. We see for the regions in lobules VIIb and VIIIb a comparable pattern for the eccentricity progression as was previously found with the pRF model (A) for both MW and RS (B). Interestingly, for region OMV we see an increase in the progression of eccentricity that extends to much higher eccentricities than in the original pRF-based analysis [*t*_(173)_ = 41.55, *p* << 0.001 (MW), *t*_(173)_ = 42.51, *p* << 0.001 (RS)]. This increase is particularly striking in the MW experiment, where the blue line lies above and shows a steeper increase of eccentricity over the cerebellar surface than in the RS experiment.

The repeated split-half analysis shows that the observed eccentricity gradients in all the topographic areas apart from left VIIb RS are significantly stable. OMV: *p* < 1 × 10^−4^ (left OMV MW), *p* = 5 × 10^−4^ (right OMV MW), *p* ≤ 1 × 10^−4^ (left OMV RS), *p* = 4 × 10^−4^ (right OMV RS). VIIb: *p* < 1 × 10^−4^ (left VIIb MW), *p* < 1 × 10^−4^ (right VIIb MW), *p* = 0.037 (left VIIb RS), *p* = 1 × 10^−4^ (right VIIb RS). VIIIb: *p* = 2 × 10^−4^ (left VIIIb MW), *p* < 1 × 10^−4^ (right VIIIb MW), *p* < 1 × 10^−4^ (left VIIIb RS), *p* < 1 × 10^−4^ (right VIIIb RS).

We retested the preferences of lower visual field bias (*y*) and ipsilaterality (hemispheric preference) as they were found in the previously found topographic areas (van Es et al., 2019) for the CF models that were estimated on RS and MW data using a *t* test. We find a lower visual field bias in all these areas, for both experiments. For the MW experiment we found ipsilaterality in all these areas, for the RS experiment only in OMV. Lower visual field bias: *t*_(173)_ > 11.48, *p* << 0.001 (tested for left and right OMV, VIIb, and VIIIb for both MW and RS).

Ipsilaterality: OMV: *t*_(173)_ = 6.90, *p* << 0.001 (OMV MW), *t*_(173)_ = 5.87, *p* << 0.001 (OMV RS). VIIb: *t*_(173)_ = 3.34, *p* = 0.001 (VIIb MW), *t*_(173)_ = 0.26, *p* = 0.79 (VIIb RS). VIIIb: *t*_(173)_ = 5.85, *p* << 0.001 (VIIIb MW), *t*_(173)_ = 0.62, *p* = 0.54 (VIIIb RS).

### New topographic organized area in Crus II

**Figure 4.**
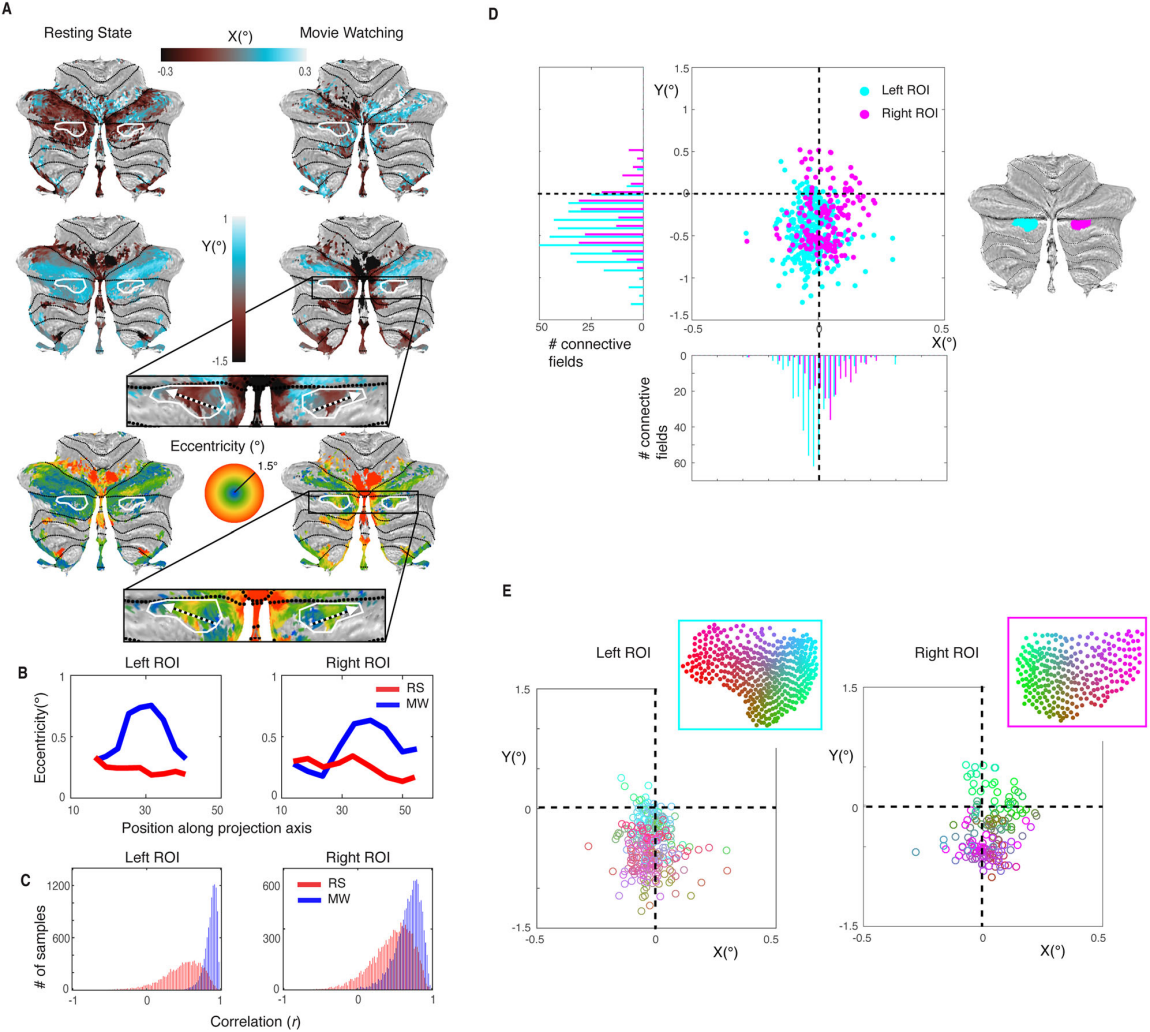
New cerebellar cluster with topographic visual organization during MW. ***A***, Both the eccentricity and the *y* parameters of the CF show a reversal in the white outlined area (blue-green-blue in eccentricity, red-yellow-red in *y*), indicating a dependency of visual field position and position on the cerebellar surface. ***B***, Eccentricity values sampled in the SUIT map projected along the direction indicated with the arrow in the zoomed-in map in ***A***. This confirms the reversal as it is visible on the SUIT map. ***C***, Histogram of the results from the repeated half-split correlation values (*r*) of the eccentricity progressions in ***B*** show that the progression of the eccentricity gradient is (more) robust in the MW experiment. ***D***, Distribution of the CF centers shows an ipsilateral visual field representation (pink right, blue left, in histogram of *x*) and a bias for the lower visual hemifield (negative *y*-axis). ***E***, Position in the visual field for every cerebellar location of the SUIT map represented using a colormap based on the position on the cerebellar surface. This shows how gradients of visual field preference are topographically connected to gradients on the cerebellar surface.

Visualizing the positions of the CFs (*x*, *y*, and eccentricity) on the surface of the cerebellum (Fig. 4*A*) revealed a novel visually organized area in the CRUS II area for the MW experiment. Visual field maps are characterized by gradual changes and reversals in the visual field over the cortex (Wandell et al., 2007). In the new area, we see a reversal in both the *y* and the eccentricity parameters of the CF in both the left and right hemisphere. The white outlines on the surfaces indicate the position of the new area. The dotted arrow indicates the direction in which we see the reversal in eccentricity. To further examine the observed reversal, we plotted the average eccentricity values of the SUIT map along the direction of the arrow (Fig. 4*B*). We see in both the left and the right ROI an increase followed by a decrease in eccentricity for the MW experiment, that we do not see in the RS experiment. To confirm the reliability of the reversal, we computed the correlation value (Pearson's *r*) using a repeated split-half analysis over subjects (Fig. 4*C*). This figure illustrates the relative stability of the eccentricity pattern in the MW experiment.

### Ipsilaterality and preference lower visual field

In Figure 4*D* we plotted the positions in the visual field (*x* and *y*) of the CFs as they were projected on the SUIT surface in the visual field. Looking at the distribution of the CF centers (Fig. 4*D*), we see both for the left and right ROI an ipsilateral visual field representation (histogram of *x*) similar to known visually organized clusters in the cerebellum [Brissenden et al., 2018; van Es et al., 2019; *t*_(173)_ = 2.89, *p* = 0.0044 (left MW vs right MW)]; ipsilaterality is also confirmed by a test for hemispheric preference (*t*_(173)_ = 3.98, *p* < 0.001). Furthermore, we see a preference for the lower visual hemifield (histogram of *y*) in the new cluster. The nodes in the cerebellum respond more to the lower part of the visual field [*t*_(173)_ = 8.13, *p* << 0.001 (left MW), *t*_(173)_ = 10.64, *p* << 0.001 (right MW)]. This again agrees with previously reported properties of topographic organizations in the cerebellum (van Es et al., 2019) that shows a bias for a lower visual field representation.

### Progression of visual field position

In the new cluster where we see a reversal in the eccentricity gradient, we also see that the CFs show a bias toward the lower part of the visual field (as highlighted in Fig. 4*D*). This bias might lead to an inability to reveal changes in position, dependent on traditional parameters such as eccentricity or polar angle. To further investigate the relation between the position on the cerebellar surface and the associated position in the visual field, we plotted the positions in the visual field using a colormap that is based on its associated position on the cerebellar surface (Fig. 4*E*). Using this visualization method, we see a gradual color change over the positions in the visual field that represents the dependency of the position on the surface with the position in the visual field irrespective of a point of reference in the visual field. This confirms our finding that the change in visual field position is based on eccentricity. It illustrates the finding of a new visual topographic area where position preferences change gradually over the cortical surface.

### Location of the new topographic area

**Figure 5.**
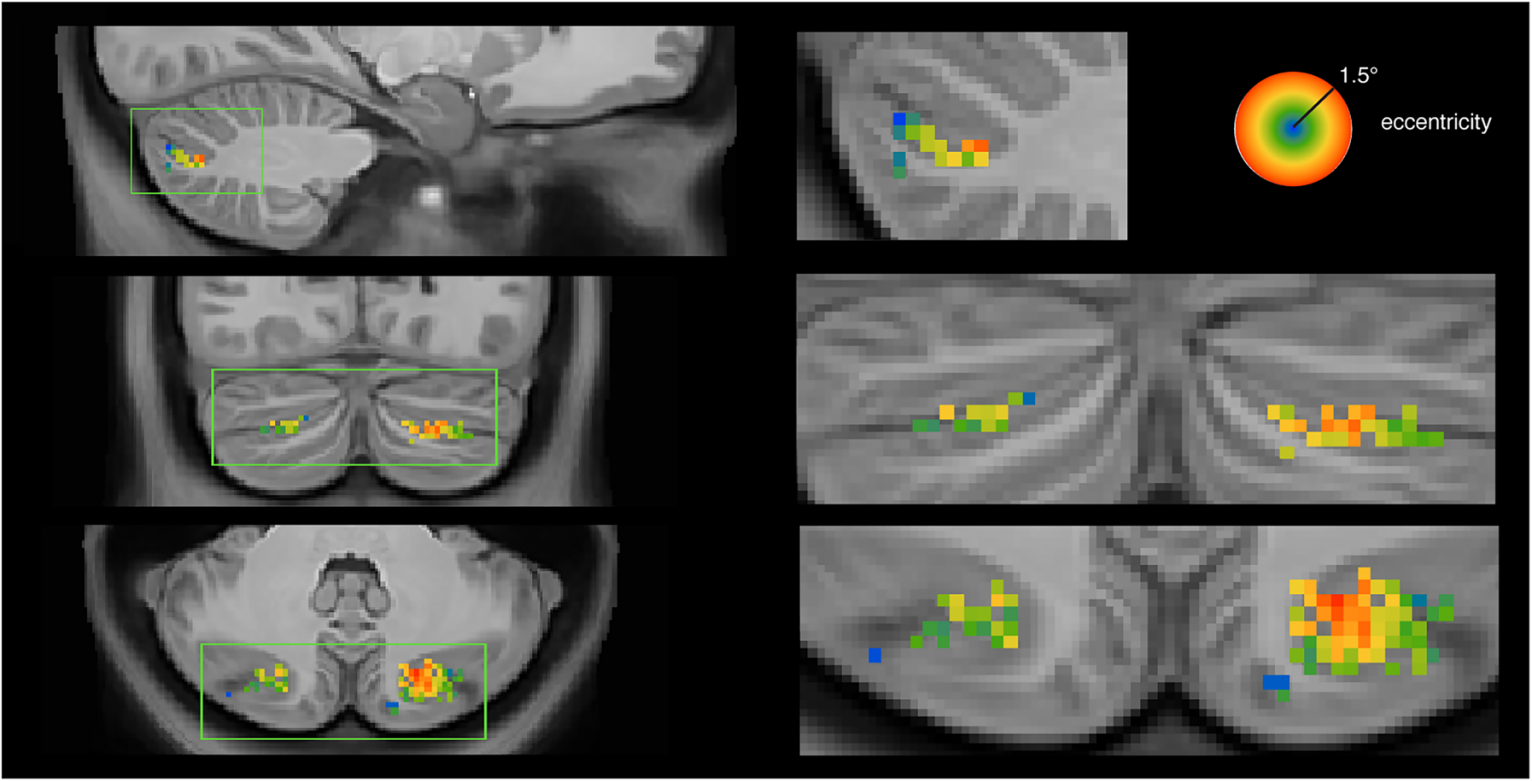
Eccentricity parameter of the new topographic area presented in the volume view.

Figure 5 shows the location of the new topographic area in the 3D volumetric space. This alternative visualization illustrates the gradual eccentricity progression of visual field preference (compare Fig. 4*A*).

**Figure 6.**
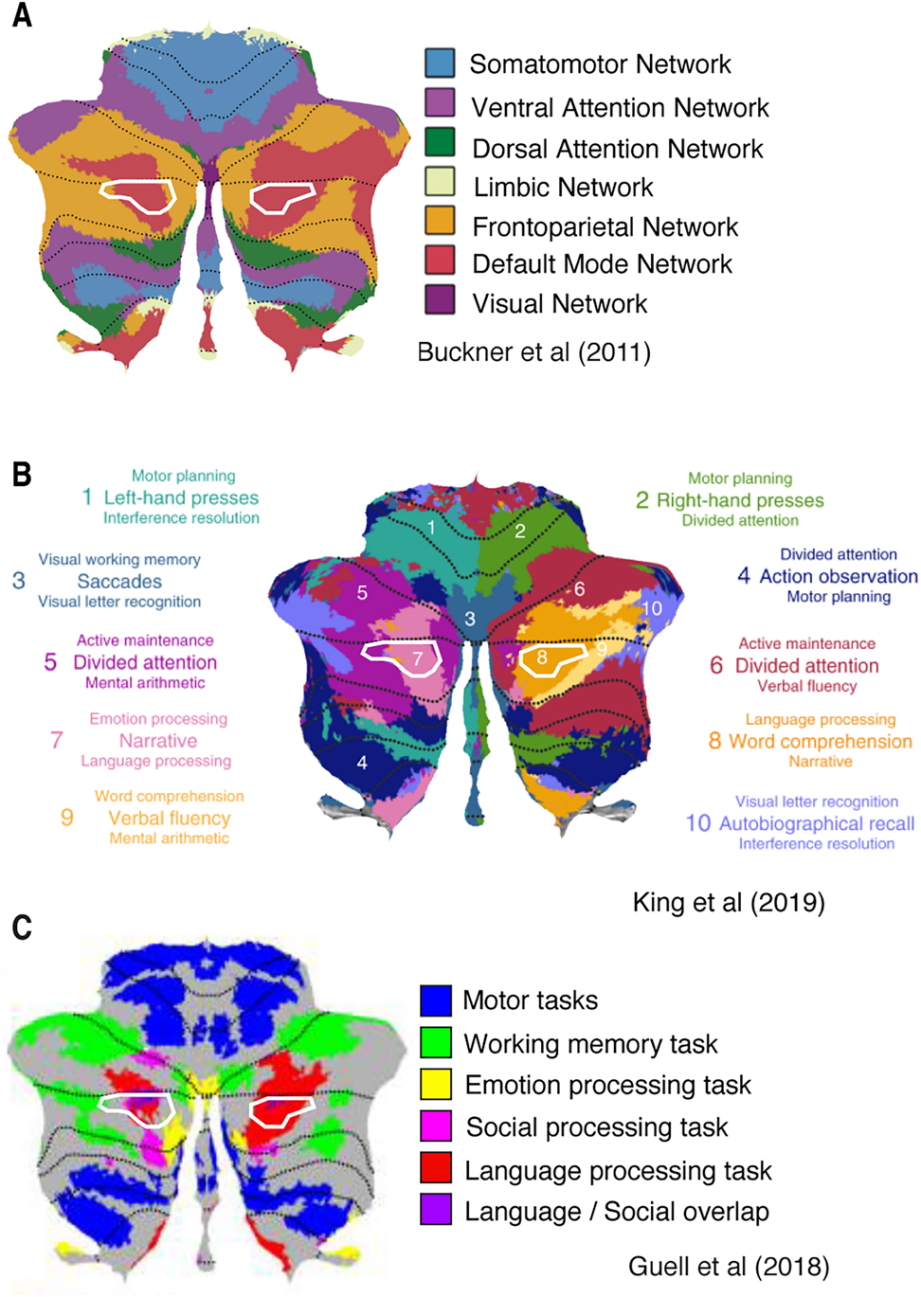
The location of the new topographic region overlaid on three different functionally defined atlases. ***A***, Shows that the region is part of the default mode network (Buckner et al., 2011). The region shows overlap with areas previously associated with emotion, language processing and social processing (Guell et al., 2018; King et al., 2019; ***B***, ***C***).

What is the cognitive role of this new visually selective area, located in the CRUS II area of the cerebellum? Previous studies have assigned specific functionality to this region. We outlined the new topographic area on top of three different functionally defined atlases of the cerebellum (Fig. 6). We chose the functional connectivity-based Buckner atlas (Buckner et al., 2011), one atlas based on a large battery of functional localizer tasks (King et al., 2019) referred to as the King atlas, and one atlas based on a more limited set of cognitive tasks from the HCP project (Guell et al., 2018), referred to as the Guell atlas.

A comparison with the Buckner atlas places the new CRUS II area in the default mode network (A). Based on the King atlas, we see that there is overlap with regions involved in emotion and language processing (B). The overlap with a region involved in language processing is also confirmed by the Guell atlas, including social processing (C).

## Discussion

We investigated the cerebellum's visual topographic organization, and the way this topographic organization is flexibly evoked dependent on cognitive state. We used a topographic connectivity model that quantifies the parameters of the so-called V1 connective field for every cerebellar voxel. This analysis depends only on ongoing signal fluctuations and is therefore not tied to any experimental design. This means we can estimate visual topographic connectivity from both movie watching and resting state experiments and directly compare between these. Our encoding model-based analysis of functional connectivity replicated earlier findings of multiple visual topographic maps in the human cerebellum. Additionally, it allowed us to reveal and chart an additional, novel visual topographic map in Crus II, thought to be part of the default mode network regions of the cerebellum.

### V1 as a source region of the CF model

Our approach to modeling topographic connectivity using single Gaussian connective fields involves a number of trade-offs and interpretational caveats. Firstly, we want to be clear that our approach is intended to find evidence of visual spatial processing in a general sense. Its findings should not be interpreted as evidence of direct connections between V1 and target regions or of these regions performing similar visual functions to V1. CF modeling is blind to which cognitive factors drive localized V1 BOLD responses: in principle, any combination of bottom-up visual processing, attentional selection, mnemonic effects, etc., could drive CF modeling results. This ambiguity underscores the need to compare CF results across experimental conditions that vary the cognitive factors at work, comparisons we undertake in the present work.

Indeed, it is possible and, in many cases, likely that alternative source regions would capture higher proportions of target signal variance and/or provide more meaningful insights as to a target region's topographic organization. Ideally, one would extend the CF modeling framework to allow for the possibility of multiple source regions’ topographic activation patterns explaining a target voxel's responses. However, in the current fitting regime the computational complexity of this would be prohibitive and we leave this endeavor for future work. Here, we conservatively focus on V1-derived connectivity and would argue that the utility of V1 as a source region centers on the fact that (1) it contains the canonical retinotopic map, (2) the size of its surface, and (3) the anatomical stability of its retinotopic map's location across individuals. All of these factors help to increase the interpretability and stability of our findings.

### Performance of the CF model throughout the cerebellum

Signals throughout the brain, including the cerebellum, can be seen as reflecting topographic functional connectivity to V1, during both rest and movie watching. Specific regions, however, differ in whether topographic connectivity (as quantified by cross-validated prediction performance) is stronger during MW or RS. During rest, the CF model better captures the timeseries of the cerebellum's default mode network and frontoparietal network regions. This is in line with previous findings showing a similar pattern of results for cerebral cortex (Knapen, 2021). Conversely, during movie watching, we find stronger CF model performance in the cerebellar areas that have previously been shown to have a visual topographic organization (van Es et al., 2019). These parallels point to a highly congruous high-level organization shared by both cerebral cortex and cerebellum, in terms of both the interplay between default mode network and sensory regions, and the shared sensory-topographic organization of these regions.

### Eccentricity preference in OMV

For every cerebellar voxel, the CF model derives the region of V1 surface, and by extension, visual space that it best corresponds to. From these estimates, we derived the visual field parameters such as eccentricity and polar angle. On the whole, this analysis confirmed the previously found properties of these cerebellar visual regions, including their ipsilateral visual representations and lower visual field bias. Interestingly, an earlier finding of polar angle maps in OMV showed that these maps lacked an expected orthogonal gradient of eccentricity: OMV pRFs showed a very strong foveal bias (van Es et al., 2019). Contrary to these earlier findings based on a pRF model fit on retinotopic mapping data, we do see an increase in the progression of eccentricity for the CF model. The eccentricity gradient is visible both in the MW and the RS experiment but is more pronounced in the MW experiment. As shown previously in cerebral cortex, different cognitive states evoke a topographic visual organization with visual field representations that differ primarily in the foveal bias of their eccentricity distribution (Knapen, 2021).

These differences in model parameters between experiments can provide information about the function of the region where these differences are found. We first focus on region OMV, known for its role in oculomotor behavior and spatial attention (Voogd et al., 2012; King et al., 2019) and where the direction preference of saccades were found to be anatomically organized (Sedaghat-Nejad et al., 2022). The pRF model was fit on data from a standard retinotopic mapping experiment with fixation task and confirmed task adherence. In contrast, during the MW experiment, participants could express a much wider range of oculomotor behaviors. We believe that this wider oculomotor range likely expanded the eccentricity range of visually related responses in OMV. In turn, this interpretation also means that it is likely that it was the oculomotor signal related to maintaining fixation in the retinotopic mapping experiment that drove their strong foveal bias. This interpretation is congruent with the fact that during RS, we find a smaller range of eccentricity values in OMV, which nonetheless is larger than the foveal eccentricity of the retinotopic mapping experiment.

### New topographic organized region found only in MW experiment

In Crus II of the cerebellum, we found a novel visually organized region based on topographic connectivity. The region shows a double representation of the lower visual field and reveals similar properties as the retinotopic maps that were found previously in the cerebellum using pRF modeling of visual field mapping data (van Es et al., 2019). The Crus II regions were found both in the left and right hemisphere, with a similarly oriented reversal of their eccentricity gradient. Reversals in visual field position preferences on the cortex are used to delineate visual field maps in cerebral cortex (Wandell et al., 2007) and the cerebellum (van Es et al., 2019). Like other visually organized cerebellar regions, these new regions show an ipsilateral preference for visual field positions. Lastly, they show a preference for the lower visual field, a property again parallel to established cerebellar visual field representations (van Es et al., 2019).

The visual organization we identify in Crus II exists during both RS and MW experiments. For both models we see a topographic improvement (Δ*R*^2^) over the nontopographic null model. This means that both models show spatially specific topographic connectivity. However, the way in which this region represents visual space differs between these experiments. The reversal in the eccentricity parameter is only visible in the MW experiment.

Whereas visual representations exhibit a strong foveal bias in rest, the movie watching experiment evokes a broader distribution of eccentricity preferences. This increased range of visual field preferences during movie watching permits the visualization and quantification of the topographic maps of visual space in this region.

The new topographically organized region is located in the Crus II of the cerebellum and has (to our knowledge) not been previously defined as being visually selective. We now show that the region is involved in the processing of visual information. Previous findings have established that this specific region is activated during emotion, language, and social tasks (Guell et al., 2018; King et al., 2019, 2023). In line with this pattern of task activations, resting state functional connectivity analyses show this region to be part of the default mode network (DMN; Buckner et al., 2011). The DMN is suggested to play a role in high-level cognition such as mind wandering (Christoff et al., 2009; Raichle, 2015), memory formation (Mayer et al., 2009; Foster et al., 2012; Lee et al., 2017; Sormaz et al., 2018), and the processing of transmodal information (Buckner and Krienen, 2013; Margulies et al., 2016). More recently, visuospatial selectivity was found in the DMN of the cerebral cortex, using fMRI both in monkeys (Klink et al., 2021) and in humans (Szinte and Knapen, 2020). Also, in the DMN regions of the cerebral cortex, visual organization is most evident during movie watching as opposed to resting state (Knapen, 2021). This indicates that active cognitive engagement with visual information may be an important driving force behind visually organized representations in the DMN. This notion is reinforced by a recent finding that in nonhuman primates, the DMN is shown to be selectively activated by spatial and attentional shifts (Arsenault et al., 2018).

What can be the reason for this overlap between visual-sensory and high-level cognitive sensitivity in Crus II and other DMN regions? Engagement with a dynamic, naturalistic stimulus such as a video segment implies the performance in a hierarchy of tasks, ranging from simple facial recognition and shifts of gaze and spatial attention, to narrative understanding, social cognition, and theory of mind. For instance, the successful estimation of actor intent from dynamic facial expressions in a scene depends on processing steps such as the foveation of the face and a host of retinotopically specific activations in both early visual cortex and face-selective visual regions. We tentatively argue that successful simultaneous performance at these different levels of analysis requires their intimate interaction and that these interactions might take place in regions such as Crus II. This perspective aligns with the distribution of semantic preferences in the cerebellum found during story listening (LeBel et al., 2021).

Recent work has shown that a naturalistic stimulus paradigm outperforms resting state in terms of linking functional connectivity measures to trait-level behavior in cognitive and emotional domains. The fact that movie watching but not resting state reveals cerebellar topographic maps also indicates that we cannot depend solely on resting state experiments to reveal the full spectrum of functional connectivity entertained by the brain. Our findings indicate that especially at the level of detail of topographic maps, within which the content of our experiences is encoded, it is necessary to evoke rich, multisensory experiences in our subjects.

In sum, we reveal new spatial properties of the visual topographic organization in the cerebellum. Where conventional connectivity studies only highlight the functionally connected regions, CF modeling allows us to reveal spatial properties of the topographic organization in high detail for multiple conditions, including naturalistic conditions. We show that with a more naturalistic experiment, properties of the topographic organization in the cerebellum are revealed that otherwise go unnoticed. This leads to a better understanding of the role of the cerebellum in the processing and integration of visual information.
